# The impact of obesity on rates of post-operative CSF leak following endoscopic skull base surgery: results from a prospective international multi-centre cohort study

**DOI:** 10.3389/fendo.2024.1353494

**Published:** 2024-06-05

**Authors:** CRANIAL Consortium

**Keywords:** pituitary adenoma, endoscopic surgery, expanded endonasal approach, CSF leak, obesity

## Abstract

**Aims:**

Post-operative CSF leak is the major source of morbidity following transsphenoidal approaches (TSA) and expanded endonasal approaches (EEA) to lesions of the sella turcica and the ventral skull base. There are conflicting reports in the literature as to whether obesity (BMI ≥30) is a risk factor for this complication. We aimed to evaluate data collected as part of prospective multi-centre cohort study to address this question.

**Methods:**

The CRANIAL (CSF Rhinorrhoea After Endonasal Intervention to the Skull Base) study database was reviewed and patients were divided into obese and non-obese cohorts. Data on patient demographics, underlying pathology, intra-operative findings and skull base repair techniques were analysed.

**Results:**

TSA were performed on 726 patients, of whom 210 were obese and 516 were non-obese. The rate of post-operative CSF leak in the obese cohort was 11/210 (5%), compared to 17/516 (3%) in the non-obese cohort, which was not statistically significant (χ^2 =^ 1.520, p=0.217). EEA were performed on 140 patients, of whom 28 were obese and 112 were non-obese. The rate of post-operative CSF leak in the obese cohort was 2/28 (7%), which was identical to the rate observed in the non-obese cohort 8/112 (7%) Fisher’s Exact Test, p=1.000). These results persisted following adjustment for inter-institutional variation and baseline risk of post-operative CSF leak.

**Conclusion:**

CSF leak rates following TSA and EEA, in association with modern skull base repair techniques, were found to be low in both obese and non-obese patients. However, due to the low rate of post-operative CSF leak, we were unable to fully exclude a small contributory effect of obesity to the risk of this complication.

## Introduction

Tumours of the sellar region are now primarily approached via an endoscopic endonasal transsphenoidal approach (TSA), and more recently the indications for endoscopic endonasal surgery have broadened to include extrasellar lesions of the ventral skull base, which can be resected via an expanded endonasal approach (EEA) (1–4). Although these less invasive approaches are demonstrably associated with improved neurological outcomes following surgery when compared to more traditional transcranial approaches, the significant shortcoming associated with their use is the occurrence of post-operative CSF leak (5–9). However, data from a recent national level study conducted in Italy and a systematic review of the literature have demonstrated that the incidence of this complication following EEA has declined over in time, in parallel with increasing surgical experience and improved skull base reconstruction (4, 10). Nevertheless, this complication is a frequent cause for post-operative re-admission, often requires operative re-intervention and can lead to significant morbidity in the form of pneumocephalus and/or meningitis (11, 12).

It has been suggested in a number of studies that a pre-operative diagnosis of obesity increases the risk of post-operative CSF leak. There are several series describing this association with TSA (13–15), as well with more the more extensive EEA (16–18). Proponents of this theory have hypothesised that obese patients are at higher risk of this complication due to increased intra-abdominal pressure, which secondarily leads to decreased venous drainage from the head and neck and elevated ICP (13). Hormonally driven pathophysiological mechanisms may also be at play: obese individuals have demonstrably increased expression of cortisol within the choroid plexus, which demonstrably increases CSF production and has been implicated in the pathophysiology of idiopathic intracranial hypertension (19). Contrary to the publications citing a link between obesity and post-operative CSF leak, other series including large numbers of patients have not reported any association between obesity and post-operative CSF leak (20–23). However, all of the aforementioned studies are limited by the fact that they are retrospective, single-centre, self-adjudicated case series. Considering the significant clinical and financial implications of a post-operative CSF leak, the identification of a modifiable pre-operative risk factor for this complication is of considerable significance and there is a requirement for well-designed, robust studies to examine this question (11, 24).

CRANIAL (CSF Rhinorrhoea After Endonasal Intervention to the Skull Base) was a prospective multi-centre cohort study run across the UK and Ireland that sought to document the variety of skull base repair protocols employed following endoscopic skull base surgery in a prospective, non-biased manner, as well as to establish the rates of post-operative CSF leak and the risk factors associated with this complication. Using the data generated from this study, we reported an overall CSF leak rate of 3.9% following TSA and 7.1% following EEA (25–27). On multivariate logistic regression analysis, the only factors associated with post-operative CSF leak were revisional surgery and the presence of an intra-operative CSF leak, while the use of tissue sealant was found to be protective. In view of the discrepant reports in the literature regarding the link between obesity and post-operative CSF leak, we elected to interrogate the study data to assess for differences in skull base repair technique in obese patients, as well as to determine if these individuals were at greater risk for post-operative CSF leak following TSA and EEA.

## Methods

A multicentre, prospective, observational cohort study design was conducted across 30 tertiary care neurosurgical centres representing 91% (29/32, of adult neurosurgical centres performing endonasal skull-base neurosurgery in the UK and Ireland). One paediatric centre was included, whilst others provided both adult and paediatric services. The study period included 6 months of consecutive case recruitment (10/08/20–10/02/21) and 6 months of follow-up (10/02/21–10/08/21). Cases included adult and paediatric patients undergoing TSA for sellar tumours and EEA for skull base tumours. TSA was defined as surgical access to the sella alone (transsphenoidal) whilst EEA was defined as acquiring surgical access to an area not limited to the sella (e.g., transplanum or transclival) (28–30).

Each centre registered the project as a service evaluation with appropriate local approvals. Local study teams consisted of consultant lead(s) with overall project

responsibility, with trainee lead(s) and student lead(s) for data collection via a secure web-based central database (Castor Electronic Data Capture) (see **Supplementary Data** for a full list of participating centres and contributors). Data were collected as per the previously published protocol and were confirmed with the operating surgeon prior to submission (31). A randomly selected cohort of 10% of patients from each centre were screened for accuracy by an independent auditor following final data submission. The Kelly grading system was used to grade intraoperative CSF leak when relevant (32). Primary outcomes were: (1) methods of skull-base reconstruction, and (2) postoperative CSF rhinorrhoea biochemically confirmed or requiring intervention (CSF diversion and/or operative repair) occurring at any time during the 6 month follow-up period. Obesity was defined as a body mass index (BMI) of ≥30kg/m^2^ at the time of surgery.

### Statistical methods

Descriptive statistics were used to summarise baseline characteristics (demographic, tumour, and operative characteristics) and surgical outcomes. Between group differences in categorical variables were compared using the Fisher’s exact test or the χ^2^ test, as appropriate. To account for differences in case mix manifesting as variation in baseline risk for CSF leak, we additionally fitted mixed effects logistic regression models with a random intercept for each centre and a fixed effect examining the influence of obesity, with varying model specifications (33). From these models, we report the odds ratio with 95% confidence interval and the marginal effect, which represents the expected change in absolute risk in patients with versus without obesity (34). Finally, we examined whether obesity was associated with a higher grade intraoperative CSF leak using proportional odds regression models to determine the relationship between Kelly leak grade and obesity, from which we report the common odds ratio (OR) with 95% confidence interval (35). All statistical analyses were performed in SPSS v25 (IBM Corp, USA). This study was performed according to the STROBE guidelines (36).

## Results

### Transsphenoidal approaches

During the study period, TSA were performed on 726 patients, of whom 210 were obese and 516 were non-obese. **Table 1** summarises the demographic and clinical details of each group of patients. Both groups were well matched with respect to age and gender. Regarding the underlying pathology, the only significant intergroup difference identified was a significantly higher proportion of functioning adenomas in the obese group when compared to the non-obese group (89/210 (42%) *vs*. 160/516, (31%), χ^2^ = 9.118, p=0.002), with a corresponding lower proportion of non-functioning adenomas (101/210 (48%) *vs*. 309/516 (59%) χ^2^ = 8.438, p=0.003). This is likely as a consequence of the expected higher proportion of patients with Cushing’s disease in the obese cohort (30/210 (14%) *vs*. 37/516 (7%), χ^2^ = 7.320, p=0.007). Otherwise, there was no significant difference in the proportion of patients with tumours >1cm in diameter (167/210 (80%) *vs*. 440/516 (85%) χ^2^ = 3.597, p=0.058) or in the proportion of patients undergoing revisional surgery (30/180 (16%) *vs*. 68/491 (13%), χ^2^ = 0.838, p=0.359) between the obese and non-obese group. Moreover, the proportion of patients in whom an intra-operative CSF leak was observed was similar in both groups (63/210 (30%) *vs*. 151/516 (29%), χ^2^ = 0.038, p=0.843), as was the distribution of patients with a high-grade (Kelly Grade 3) intra-operative CSF leak (1/210 (<1%) *vs*. 4/210 (<1%), χ^2^ = 0.195, p=0.658).

**Table 1:** Table demonstrating the key characteristics of obese and non-obese patients who underwent a trans-sphenoidal approach to intra-sellar pathology.

	Obese (BMI >30kg/m^2^) (n=210)	Non-Obese (BMI ≤30kg/m^2^) (n=516)
Baseline Characteristics		
Median Age (IQR)	51 (22)	54 (22)
Female (%)	102 (49)	269 (52)
Surgical Pathology		
Non-functioning Pituitary Adenoma (%)*	***101 (48)***	***309 (59)***
Functioning Pituitary Adenoma (%)*	***89 (42)***	***160 (31)***
Rathke’s Cleft Cyst (%)	7 (3)	19 (4)
Other Pathology (%)	13 (6)	48 (9)
Diameter >1cm (%)	167 (80)	440 (85)
Revision Surgery (%) †	30 (16)	68 (13)
Intra-Operative CSF Leak		
Grade 0 (%)	147 (70)	365 (71)
Grade 1 (%)	42 (20)	89 (17)
Grade 2 (%)	9 (4)	45 (9)
Grade 3 (%)	1 (<1)	4 (<1)
Leak present, Grade Unknown (%)	11 (5)	13 (3)
Skull Base Repair Techniques		
Dural Replacement (%)	57 (27)	139 (27)
Tissue Graft (%)	62 (29)	159 (31)
Synthetic Graft (%)	59 (28)	145 (28)
Tissue Sealant (%)	147 (70)	327 (64)
Haemostatic Agent (%)	135 (64)	304 (59)
Vascularised Flap (%)	37 (17)	79 (15)
Nasal Packing (%)	156 (74)	363 (70)
CSF Diversion (%)	9 (4)	20 (4)

† Data on whether surgery was primary or revision was missing in 55 cases.

Bold italic font and * indicates statistically significant intergroup comparison.

**Figure 1:**
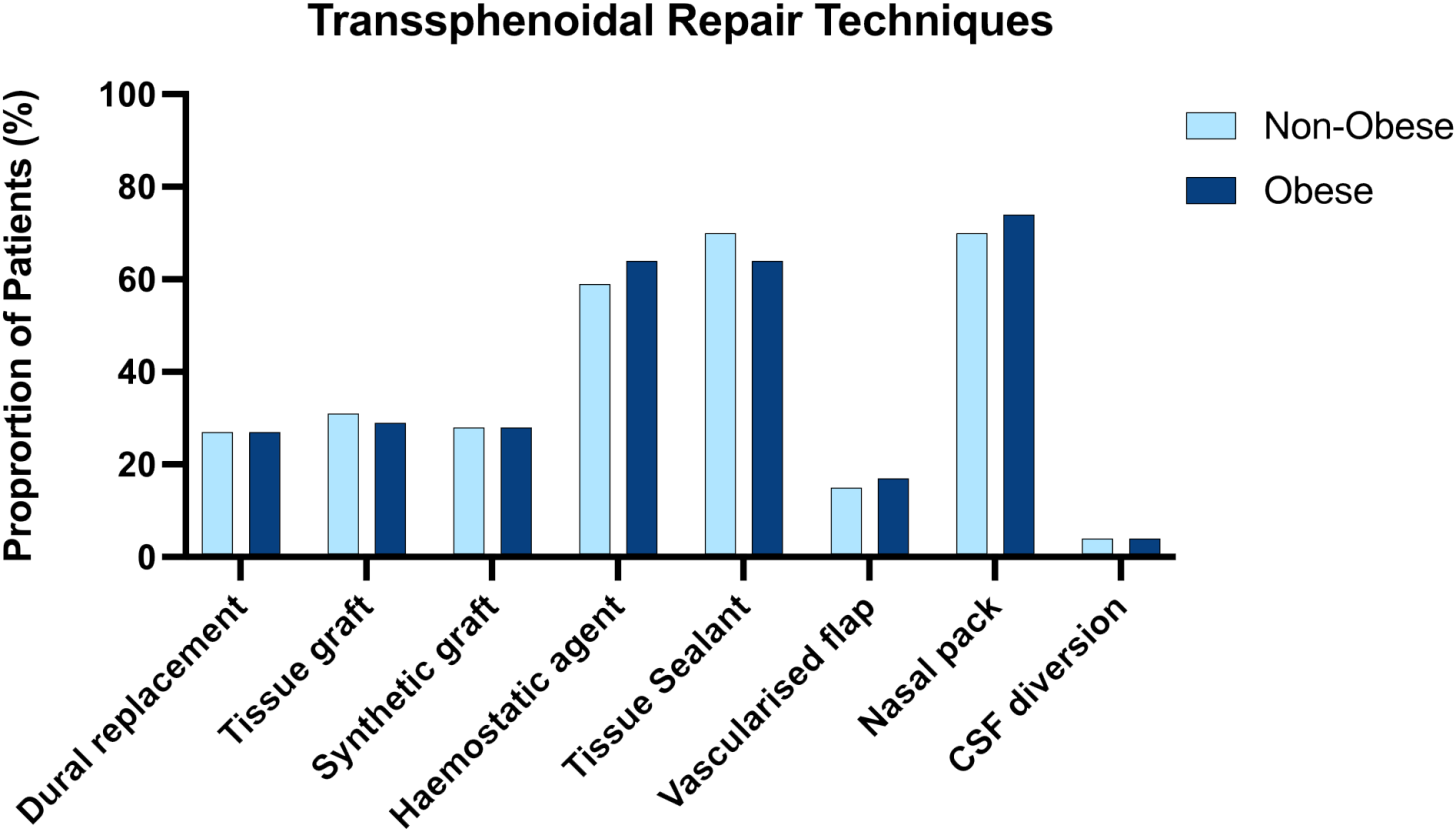
Side by side column graph indicating the frequency of skull base repair techniques employed per centre for obese and non-obese patients undergoing trans sphenoidal approaches. There was no significant difference in the frequency of any repair method when obese and non-obese patients were compared.

The techniques employed for skull base repair in the obese and non-obese groups were similar, with no apparent inter-group differences in the methods of skull base repair that were employed (**Figure 1**)

The rate of post-operative CSF leak was greater in the obese cohort but this did not reach statistical significance (11/210 (5%) *vs*. 17/516 (3%), χ^2 =^ 1.520, p=0.217). The median time to the occurrence of CSF leak in the obese cohort was 2 days (range 1-7 days) and 2 days (range 1-17 days) in the non-obese cohort.

### Expanded endonasal approaches

During the study period, EEA were performed on 140 patients, of whom 28 were obese and 112 were non-obese. **Table 2** summarises the demographic and clinical details of each group of patients. Both groups were well matched with respect to age and gender. There was an even distribution of underlying pathology when the two groups were compared. With regard to pathology addressed exclusively through EEA, the proportion of chordomas (2/28 (7%) *vs*. 13/112 (12%), Fisher’s Exact Test, p=0.738), craniopharyngiomas (5/28 (18%) *vs*. 29/112 (29%), Fisher’s Exact Test, p=0.245) and meningiomas (6/28 (21%) *vs*. 19/112 (17%), χ^2 =^ 0.304, p=0.581) did not differ significantly between groups. Similarly, there was no significant difference in the proportion of patients with tumours >1cm (25/28 (89%) *vs*. 106/112 (95%), χ^2 =^ 1.069, p=0.301) or of patients undergoing revisional surgery (6/27 (21%) *vs*. 15/107 (13%), χ^2 =^ 1.098, p=0.295) in the obese as compared to the non-obese groups. There was also no significant difference in the proportion of patients with any grade of intra-operative CSF leak (17/28 (61%) *vs*. 62/112 (52%), χ^2 =^ 0.894, p=0.344) when both groups were compared. There was however, a significantly higher proportion of high-grade (Kelly Grade 3) intra-operative CSF leak (11/28 (39%) *vs*. 28/112 (25%, χ^2 =^ 13.099, p=<0.001) within the obese cohort.

**Table 2:** Table demonstrating the key characteristics of obese and non-obese patients who underwent an expanded endonasal approach to pathology extending beyond the sella turcica.

	Obese (BMI >30kg/m^2^) (n=28)	Non-Obese (BMI ≤30kg/m^2^) (n=112)
Baseline Characteristics		
Median Age (IQR)	55 (26)	49 (30)
Female (%)	14 (50)	65 (58)
Surgical Pathology		
Chordoma (%)	2 (7)	13 (12)
Craniopharyngioma (%)	5 (18)	33 (29)
Meningioma (%)	6 (21)	19 (17)
Non-Functioning Pituitary Adenoma (%)	8 (29)	15 (14)
Functioning Pituitary Adenoma (%)	0 (0)	13 (12)
Other Pathology (%)	7 (25)	19 (17)
Diameter >1cm (%)	25 (89)	106 (95)
Revision Surgery (%) †	6 (21)	15 (13)
Intra-Operative CSF Leak		
Grade 0 (%)	11 (39)	50 (45)
Grade 1 (%)	2 (7)	10 (9)
Grade 2 (%)	1 (4)	12 (11)
Grade 3 (%)*	***11 (39)***	***28 (25)***
Leak present, Grade Unknown (%)	3 (11)	12 (10)
Skull Base Repair Techniques		
Dural Replacement (%)	15 (54)	51 (46)
Tissue Graft (%)	13 (46)	52 (46)
Synthetic Graft (%)	11 (39)	36 (32)
Tissue Sealant (%)	25 (89)	89 (79)
Haemostatic Agent (%)	19 (68)	74 (66)
Vascularised Flap (%)	21 (75)	69 (62)
Nasal Packing (%)	23 (82)	93 (83)
CSF Diversion (%)	7 (25)	31 (28)

† Data on whether surgery was primary or revision was missing in 6 cases.

Bold italic font and * indicates statistically significant intergroup comparison.

**Figure 2:**
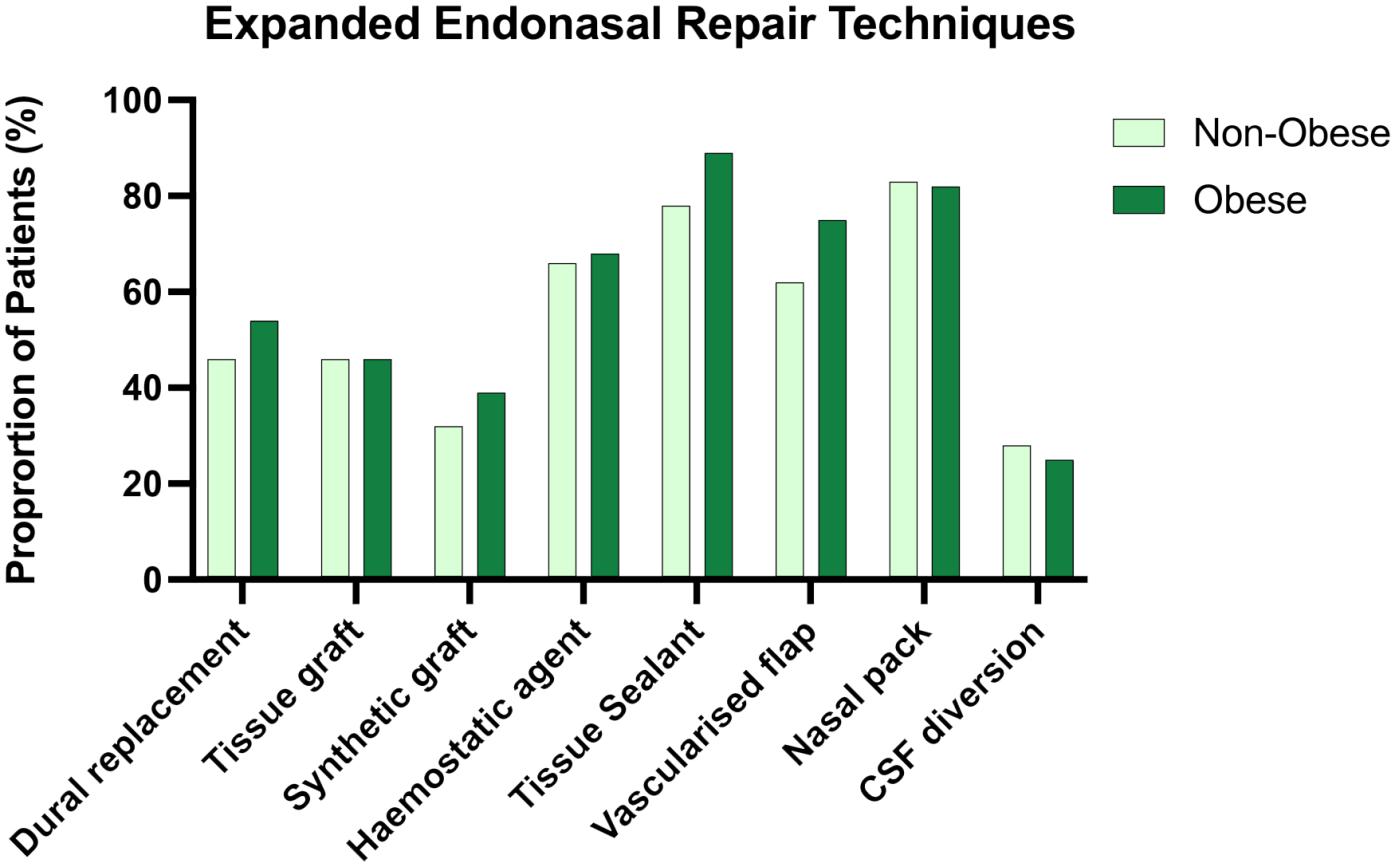
Side by side column graph indicating the frequency of skull base repair techniques employed per centre for obese and non-obese patients undergoing expanded endonasal approaches. There was no significant difference in the frequency of any repair method when obese and non-obese patients were compared.

Methods used for skull base repair appeared to be approximately distributed between obese and non-obese patients (**Figure 2**).

The rate of post-operative CSF leak in the obese cohort was 2/28 (7%), which was not significantly different to the rate observed in the non-obese cohort 8/112 (7%) Fisher’s Exact Test, p=1.000). The median time to the occurrence of CSF leak in the obese cohort was 3 days (range 1-11 days) and 3 days (range 1-6 days) in the non-obese cohort.

### Effect of case mix

Adjustment of models for between-centre variability did not influence the effect of obesity on either a univariable (OR 1.25, 95%CI 0.61 to 2.55) or multivariable basis (OR 1.6, 95%CI 0.7 to 3.7) (**Table 3**). In proportional odds models adjusted for approach, obesity was not associated with a higher grade of intraoperative leak (common OR 0.98, 95%CI 0.7 to 1.38).

**Table 3:** Results of mixed effects logistic regression models, displayed as odds ratios and marginal effects, which represent the change in absolute risk for patients with versus without obesity.

Model	Odds Ratio (95% CI)	Change in risk (%, 95% CI)
Univariable	1.39 (0.68 to 2.73)	+1.39% (-1.51 to +4.3)
Case mix-adjusted^a^	1.25 (0.61 to 2.55)	+0.8% (-2 to +5.88)
Baseline-adjusted^b^	1.29 (0.62 to 2.66)	+0.68% (-2.14 to +3.5)
Multivariable^c^	1.6 (0.7 to 3.7)	+1.5% (-1.32 to +4.3)

**^a^** Model includes a random per-centre intercept.

**^b^** Model adjusted for age, sex and a random per-centre intercept.

**^c^** Model adjusted for approach, presence of an intra-operative leak and a random per-centre intercept.

## Discussion

### Key findings

This prospective cohort study of over 800 patients undergoing TSA and EEA is the first of its kind, and gathered data from the overwhelming majority of neurosurgical centres in the UK & Ireland. Utilising data collected in the course this study, we found that CSF leak rates following TSA and EEA were very low in both obese and non-obese patients. We found no significant association between obesity and the methods of skull base repair or of post-operative CSF leak. We did however, observe a trend towards more frequent use of vascularised flaps and of post-operative CSF leak in the obese cohort, and given the very low CSF leak rates we cannot entirely exclude the presence of a smaller effect. For an independent, two-tailed test for difference in proportions, we estimate that our study was adequate to demonstrate an effect size of OR 2.76. Our results therefore suggest that the effect size, if present, is likely to be smaller than an OR of 2.76 (13, 37, 38).

### Interpretation

The first study that provided evidence of a link between obesity and post-operative CSF leak following TSA was a retrospective single-centre analysis of 95 patients, operated between 2005 and 2010, with an overall post-operative CSF leak rate of 14%. The mean BMI of the entire cohort was 33.7 kg/m^2^. The authors reported a significantly higher BMI in those patients with a post-operative CSF leak when compared to those patients who did not suffer this complication (39.2 *vs* 32.9 kg/m^2^, p =0.006) (13). This finding persisted following multivariate analysis and was subsequently replicated in two other studies with a similar design (37, 38). Moreover, a significant number of studies have investigated for a correlation between increased BMI and rates of post-operative CSF leak and reported no association (20–23). It is therefore clear that there is considerable discrepancy in the literature regarding the potential impact of increased BMI on the risk of post-operative CSF leak, and our finding of no increased risk in obese patients undergoing TSA are in keeping with those from a number of others.

The literature is similarly divergent when EEA to extrasellar pathology is considered; in one of the first analyses of risk factors for CSF leak following EEA, Ivan et al. retrospectively analysed 98 operations performed for a variety of pathologies of the ventral skull base, including large pituitary adenomas, chordomas and meningiomas. Despite hypothesising that increased BMI would be associated with an increased rate of post-operative CSF leak, the authors were only able to demonstrate a statistically significant association when those with an abnormally low and high BMI (BMI ≤18 kg/m^2^ or ≥25kg/m^2^) were amalgamated into one group with an ‘abnormal BMI’, and no significant increase in BMI was observed in those who demonstrated a post-operative CSF leak (16). A further multi-centre study of 70 patients undergoing EEA resulting in a high-flow intra-operative CSF leak found no association between obesity and post-operative CSF leaks (39). However, Torres-Bayona et al. recently published an analysis of a small number of patients with persistent post-operative CSF leak following EEA to posterior fossa tumours, and identified a BMI of ≥30 as a significant predictor of persistent CSF leak (18).

Unsurprisingly, in our cohort we demonstrated a higher rate of CSF leak in patients undergoing EEA (7%) when compared to those undergoing TSA only (3.9%)-this in keeping with previous studies in the literature demonstrating increased rates of this complication following EEA (40). Moreover, in the setting of craniofacial trauma, it has been demonstrated that more extensive bony injury and a penetrating mechanism are associated with an increased rate of CSF leak (41). The introduction of the vascularised nasoseptal flap (NSF), pedicled on the sphenopalatine artery, was transformative in the reduction of the incidence of post-operative CSF leak following EEA, which were in excess of 30% in early series (40, 42, 43). In the intervening period following the introduction of the NSF further adjuncts to skull base repair following EEA have been advocated, including the use of tissue sealants, multiple layers of autologous fascia lata, autologous fat graft and nasal packing, although none have demonstrated such a dramatic impact on the rate of post-operative CSF leak as the use of the NSF (44–46). In an effort to further reduce the incidence of post-operative CSF leak, the use of prophylactic lumbar drainage has been advocated: a single centre randomised controlled trial (RCT) of post-operative lumbar drainage demonstrated a significant reduction in the rate of post-operative CSF leak when compared to the cohort randomised to no prophylactic lumbar drainage (8% *vs* 21%) (7). Interestingly, in our original analysis of this dataset we did not find the use of prophylactic lumbar drainage to be associated with a decreased rate of post-operative CSF leak, although our study was conducted >2 years following the publication of the aforementioned RCT, and it may have been the case that the cases felt to be at higher risk for post-operative CSF leak were more likely to have prophylactic lumbar drains placed (26). As EEA have been employed for increasing complex pathologies, encompassing both the sagittal and coronal planes of the skull base, it has been suggested that optimal results are obtained when neurosurgeons and otolaryngologists collaborate in a multidisciplinary fashion to optimise the results of surgery (47, 48). Although we cannot provide data that directly supports this assertion, in our initial analysis of this study, we observed that a neurosurgeon and an otolaryngologist were present for 90/140 of the EEA cases, suggesting that there is significant cross-specialty involvement in the majority of these cases in the UK & Ireland.

Previous work using machine learning driven analysis to analyse our prospective multicentre dataset suggested an increased rate of post-operative CSFR in patients of BMI > 30 when using the TSA approach (49). This suggests that if a relationship between obesity and CSFR exists, it is likely complex and non-linear, with possible confounding factors. This is in contrast to our findings in this study, which did not establish a relationship using in-depth traditional statistical methods, but is arguably more interpretable. Therefore, future studies should consider both forms of analysis, using larger prospective multivariate longitudinal (and ideally multimodal) datasets.

### Limitations and generalisability

The prospective, multi-centre nature of this study, which utilised semi-independent data collectors and consecutive case accrual significantly lowers the risk of biased, unrepresentative results. However, this study is subject to a number of limitations; Firstly, the number of obese patients undergoing EEA was relatively small and therefore there is an increased chance of a type 2 statistical error. However, the data collected represent six months of operative activity across two countries with a combined population of ~75 million and the low numbers of obese patients undergoing EEA reflect the rarity of the pathology necessitating this approach. Secondly, the data upon which this study is based are purely observational, and we were therefore unable to attribute causation to any factors that may have been associated with the occurrence of post-operative CSF leak. While we performed sensitivity analyses using varying assumptions to attempt to partially account for confounding, this is an observational study and thus our analysis cannot account for the presence of unmeasured confounding. As the outcome was rare, the number of observed events was small which made it impossible to fit more complex models while retaining model robustness, as this limited the acceptable degrees of freedom (PMID 29292533). Finally, despite the large catchment population these results were obtained from two European countries and only one paediatric centre was included, which may limit the generalisability of our results.

## Conclusion

In this prospective multi-centre study of over 800 patients, we did not observe an increased risk of post-operative CSF leak following EEA or TSA in association with obesity. Due to the extremely low rates of CSF leak, we were unable to fully exclude a minor contribution of obesity towards the development of this complication.

## Data availability statement

The raw data supporting the conclusions of this article will be made available by the authors, without undue reservation.

## Ethics statement

Ethical review and approval was not required for the study on human participants in accordance with the local legislation and institutional requirements. Written informed consent for participation was not required for this study in accordance with the national legislation and the institutional requirements. for the studies involving humans because Ethical review and approval was not required for the study on human participants in accordance with the local legislation and institutional requirements. Written informed consent for participation was not required for this study in accordance with the national legislation and the institutional requirements. The studies were conducted in accordance with the local legislation and institutional requirements. The ethics committee/institutional review board also waived the requirement of written informed consent for participation from the participants or the participants’ legal guardians/next of kin because Ethical review and approval was not required for the study on human participants in accordance with the local legislation and institutional requirements. Written informed consent for participation was not required for this study in accordance with the national legislation and the institutional requirements.

## Author contributions

CRANIAL Consortium: Conceptualization, Data curation, Formal analysis, Investigation, Methodology, Project administration, Software, Supervision, Validation, Writing – original draft, Writing – review & editing.

## Group members of CRANIAL Consortium

Please see **Supplementary Material**
